# An Old Concept Achieved

**Published:** 1990-03

**Authors:** R C L Feneley

**Affiliations:** President Bristol Medico-Chirurgical Society


### Editorial

### AN OLD CONCEPT ACHIEVED

The launch of a new medical journal is an exciting event, inevitably associated with a certain apprehension about its future role. The concept of a West of England Medical Journal offers a unique opportunity to publicise the range of medical activities that take place in this region. The celebrations however must not cloud the immense contribution of its predecessor, the Bristol Medico-Chirurgical Journal nor the efforts of all those who have ensured its continuity over the past century. Indeed it would appear that the Editor, Mr Michael Wilson has achieved at long last one of the original concepts for the journal.

The Bristol Medico-Chirurgical Society was founded in 1874 "for the Cultivation of Medicine and Surgery in all its branches". When the Society decided in 1883 to publish a medical journal, support for the journal was enthusiastically received not just in Bristol but from throughout the West of England. The front cover of the first number bore the subtitle "A Journal of the Medical Sciences for the West of England and South Wales". Professor Greig Smith was the first Editor and before he retired from that post in 1891, the journal had made a great deal of money. The Society decided to use this to start a library which was later to become incorporated into the medical library of the University of Bristol. The journal has always been an important vehicle for postgraduate education, news and events. It is interesting to note that in March 1896 a report with an X-ray picture was presented in the Journal, only 4 months after Roentgen's original demonstration of radiology. There were also case reports, some of local interest such as the girl of 22, reported by a house surgeon Dr Fenton Evans from th BRI, who jumped from the Clifton Suspension Bridge and survived because, he suggested, of "the nature of her clothes which are said to have been inflated from below and thus delayed her descent". Medical Sciences are now advancing more rapidly perhaps than ever before and thus the need for a regular update is even more pertinent. Whilst there are many specialist journals, super-specialisation tends to encourage a style of writing that is difficult for medical practitioners in other disciplines to follow. May the Journal educate rather than frustrate its readers!

Financial viability is of course essential and the journal has survived many vicissitudes over the years. A major crisis arose in 1989 but the Nuffield Nursing Homes Trust came to the rescue as a Sponsor, generously offering to fund four publications a year. As to the future, let us hope that wider support for the journal will provide the necessary security and establish a vital self-perpetuating publication.

R C L Feneley. President Bristol Medico-Chirurgical Society

